# Chlorogenic Acid Attenuates Busulfan-Induced Azoospermia in Rats: Antioxidant-Mediated Restoration of Spermatogenesis and Steroidogenesis - A Stereological and Molecular Analysis

**DOI:** 10.5935/1518-0557.20260037

**Published:** 2026

**Authors:** Farhad Koohpeyma, Samaneh Karimi

**Affiliations:** 1 Student Research Committee, Endocrinology and Metabolism Research Center, Shiraz University of Medical Science, Shiraz, Iran; 2 Department of Anatomical Sciences, School of Medicine, Abadan University of Medical Sciences, Abadan, Iran

**Keywords:** busulfan, chlorogenic acid, azoospermia, spermatogenesis, steroidogenesis, oxidative stress

## Abstract

**Objective:**

Busulfan, a chemotherapeutic alkylating agent, induces azoospermia by generating reactive oxygen species (ROS), thereby impairing spermatogenesis and steroidogenesis, and threatening fertility in cancer patients. Chlorogenic acid (CGA), a polyphenolic compound with antioxidant and anti-inflammatory properties, may protect against such reproductive toxicity. This study evaluated the effects of CGA on testicular function in a rat model of busulfan-induced azoospermia, with emphasis on spermatogenesis, steroidogenesis, and testicular architecture.

**Methods:**

Twenty-four adult male Wistar rats were randomly assigned to four groups: control, CGA (50 mg/kg, oral, for 60 days), busulfan (BUS, 40 mg/kg, single intraperitoneal dose), and BUS+CGA. Testicular function was assessed using stereological analyses (testis weight; seminiferous tubule and germinal epithelium volumes; germ and somatic cell counts), sperm parameters, serum hormone levels, oxidative stress markers (total antioxidant capacity [TAC] and malondialdehyde [MDA]), and expression of steroidogenic genes (STAR, CYP11A1, and HSD17B3) by qRT-PCR.

**Results:**

Busulfan markedly reduced testis weight, sperm quality, testosterone, TAC, and expression of steroidogenic genes, while increasing MDA and sperm abnormalities (*p*<0.05). CGA co-administration significantly improved these parameters, restoring gene expression and TAC toward control levels, reducing MDA, and enhancing testosterone, sperm count, morphology, and testicular histoarchitecture. However, sperm motility and testicular volume remained below control values (*p*<0.05). The CGA-alone group showed no significant differences from controls.

**Conclusion:**

CGA mitigates busulfan-induced azoospermia by enhancing antioxidant defenses, and improving spermatogenesis, steroidogenesis, and testicular morphology. These findings suggest that CGA may serve as a potential complementary therapy for fertility preservation in patients undergoing chemotherapy, warranting further mechanistic and clinical investigation.

## INTRODUCTION

Male infertility, affecting 10-15% of couples globally, poses a significant health challenge, driven by environmental exposures, lifestyle factors, and iatrogenic causes such as chemotherapy (Mohlala *et al*., 2023; Khalaf *et al*., 2024). Busulfan, an alkylating chemotherapeutic agent used to treat chronic myelogenous leukemia and for conditioning before hematopoietic stem cell transplantation, induces severe gonadotoxicity, resulting in azoospermia through oxidative stress, inflammation, and germ-cell apoptosis (Abarikwu *et al*., 2022; Rostami *et al*., 2022). These effects manifest as testicular atrophy, seminiferous tubule degeneration, reduced sperm count and motility, and disrupted testosterone biosynthesis due to downregulation of key steroidogenic genes, including steroidogenic acute regulatory protein (STAR), cytochrome P450 side-chain cleavage enzyme (CYP11A1), and 17β-hydroxysteroid dehydrogenase type 3 (HSD17B3) (Pu *et al*., 2023; Wu *et al*., 2024). Busulfan-induced oxidative insult, characterized by excessive reactive oxygen species (ROS), lipid peroxidation, and activation of caspase-mediated apoptotic pathways, impairs Leydig -cell function and testicular endocrine homeostasis, thereby exacerbating infertility (Eren *et al*., 2020; Abarikwu *et al*., 2022). Similar reproductive toxicity has been observed with tripterygium glycosides (TGs), which cause asthenozoospermia and reduced intratesticular testosterone, highlighting common mechanisms of chemotherapy-induced testicular damage (Chen *et al*., 2025).

Chlorogenic acid (CGA), a polyphenolic compound abundant in coffee, fruits, and vegetables, exhibits potent antioxidant, anti-inflammatory, and anti-apoptotic properties, making it a promising candidate for mitigating chemotherapy-induced reproductive toxicity (El-Khadragy *et al*., 2021; Khan *et al*., 2024). In vitro studies have demonstrated CGA’’s ability to scavenge ROS, enhance the activity of antioxidant enzymes such as superoxide dismutase (SOD), and glutathione peroxidase (GPx), and modulate survival pathways such as PI3K/AKT and MAPK in testicular cells exposed to oxidative stress (Abedpour *et al*., 2022; Khan *et al*., 2024). For instance, Abedpour *et al*. (2022) showed that CGA protected testicular cells from ionizing radiation-induced damage by reducing ROS and enhancing cell viability. In vivo, Chen *et al*. (2025) reported that CGA restored sperm motility, testosterone levels, and antioxidant defenses in rats with TG-induced asthenozoospermia, partly through modulation of MAPK signaling. Similarly, Demir *et al*. (2025) found that CGA mitigated cisplatin-induced testicular damage by upregulating Nrf2/HO-1 signaling and reducing inflammation, whereas El-Khadragy *et al*. (2021) demonstrated protective effects of CGA against arsenic-induced reproductive toxicity by enhancing steroidogenesis and reducing apoptosis in mice. Comparable phenolic compounds, such as gallic acid and ellagic acid, have shown efficacy in ameliorating busulfan-induced testicular damage by restoring spermatogonial populations, improving tubule morphology, and normalizing hormonal balance, suggesting mechanistic similarities with CGA (Abarikwu *et al*., 2022; Rostami *et al*., 2022). Additionally, CGA’s ability to upregulate STAR expression via SHP2-mediated signaling in Leydig cells supports its potential to restore testosterone production (Mu *et al*., 2022). Despite these findings, the efficacy of CGA in counteracting busulfan-induced azoospermia, particularly through modulation of steroidogenic gene expression, remains unexplored.

Given the severe gonadotoxic effects of busulfan and the lack of effective therapeutic interventions, this study investigated the protective effects of CGA in a rat model of busulfan-induced azoospermia. Using stereological analysis for quantitative morphometric assessment and molecular techniques to evaluate STAR, CYP11A1, and HSD17B3 expression, we aimed to elucidate the effects of CGA on testicular architecture, sperm quality, hormonal profiles, and underlying mechanisms. This research addresses a critical gap in the field of chemotherapy-induced infertility and provides insights into antioxidant-based strategies for fertility preservation.

## MATERIALS AND METHODS

### Animals and Experimental Design

Twenty-four adult male Sprague-Dawley rats (8-10 weeks old, 200-250 g) were obtained from the Animal Laboratory Center of Shiraz University of Medical Sciences, Shiraz, Iran. Rats were housed under standard laboratory conditions (12-h light/dark cycle, 20-22°C, 50-60% relative humidity) with ad libitum access to standard chow and water. All procedures were approved by the Animal Ethics Committee of Abadan University of Medical Sciences (Approval No. IR.ABADANUMS.AEC.1404.003). The sample size (n=6 per group) was determined on the basis of power calculations to detect significant differences (*p*<0.05, 80% power) in testicular parameters, as informed by previous studies on busulfan-induced testicular toxicity (Abarikwu *et al*., 2022). Rats were randomly assigned to four groups (n=6 per group):

- Group I (Control): Received 0.2 mL of normal saline intraperitoneally (IP) daily for 60 days.
- Group II (CGA): Received chlorogenic acid (CGA, 50 mg/kg/day, Sigma-Aldrich, St. Louis, MO, USA; purity ≥98%) (Chen *et al*., 2025), dissolved in distilled water and administered orally by gavage daily for 60 days.
- Group III (BUS): Received a single IP dose of busulfan (20 mg/kg, Sigma-Aldrich) (Rostami *et al*., 2022), dissolved in dimethyl sulfoxide (DMSO) and diluted with saline, to induce azoospermia.
- Group IV (BUS+CGA): Received a single IP dose of busulfan (20 mg/kg), followed by CGA (50 mg/kg/day, orally by gavage) for 60 days, starting concurrently with busulfan administration.

### Anesthesia and Euthanasia

After 60 days, corresponding to the rat spermatogenic cycle (Bairy *et al*., 2010), rats were anesthetized with an intraperitoneal injection of ketamine (80 mg/kg, Alfasan, Netherlands) and xylazine (10 mg/kg, Alfasan, Netherlands) to ensure deep anesthesia (Kardaş *et al*., 2023), as confirmed by the absence of the pedal reflex. Euthanasia was performed by CO₂ inhalation with a gradual fill rate of 20-30% of the chamber volume per minute, in accordance with the AVMA Guidelines for the Euthanasia of Animals (2020) and ARRIVE guidelines 2.0 (Percie du Sert *et al*., 2020; Verma *et al*., 2024). Death was confirmed by cessation of respiration and heartbeat. Blood was collected by cardiac puncture for hormone analysis (LH, FSH, and testosterone). The left and right testes were excised and weighed. The left testis was fixed in 10% neutral-buffered formalin for 24 hours for histological and stereological analyses, whereas the right testis was snap-frozen in liquid nitrogen and stored at -80°C for biochemical assays and gene -expression analysis.

### Biochemical Analysis

#### Sex Hormone Levels

Serum testosterone, luteinizing hormone (LH), and follicle-stimulating hormone (FSH) levels were quantified using commercial enzyme-linked immunosorbent assay (ELISA) kits (My BioSource, San Diego, CA, USA): Rat/Mouse Testosterone ELISA Kit (Catalog No. MBS494055), Mouse FSH ELISA Kit (Catalog No. FY-EM13638), and Mouse LH ELISA Kit (Catalog No. MBS041300), following the manufacturers’ protocols. Absorbance was measured at 450 nm using a microplate reader (Bio -Tek, Winooski, VT, USA) (Koohpeyma *et al*., 2022).

### Total Antioxidant Capacity (TAC) Measurement

Testicular tissue homogenates were prepared in phosphate-buffered saline (PBS, pH 7.4). TAC was measured using a spectrophotometric ABTS assay at 25°C. ABTS (2,2’-azino-bis (3-ethylbenzothiazoline-6-sulfonic acid)) was reacted with peroxidase and hydrogen peroxide to produce the ABTS⁺ radical, with absorbance at 600 nm. Antioxidants in the homogenate inhibited color production, and TAC was expressed as mmol/L, calibrated against a Trolox standard (Koohpeyma *et al*., 2022).

### Malondialdehyde (MDA) Measurement

MDA, a marker of lipid peroxidation, was quantified using the thiobarbituric acid reactive substances (TBARS) assay. Testes were homogenized in 1.15% KCl to prepare a 10% (w/v) homogenate. A 0.1 mL aliquot of homogenate was mixed with 0.9 mL of 1.8% sodium dodecyl sulfate (SDS), 1.5 mL of 20% acetic acid (pH 3.5), and 1.5 mL of 0.8% thiobarbituric acid (TBA). The mixture was incubated at 95°C for 60 min, cooled, and centrifuged at 4000 rpm for 10 min. The absorbance of the supernatant was measured at 532 nm using a spectrophotometer (Shimadzu, Kyoto, Japan) (Koohpeyma *et al*., 2024).

### RNA Isolation and Quantitative Real-Time PCR Analysis

Testicular tissue samples were stabilized in RNAlater (Qiagen, Hilden, Germany) for 24 h and stored at -80°C. Total RNA was extracted using Biozol reagent (bio-WORLD, Dublin, OH, USA) according to the manufacturer’’s protocol. One microgram of RNA was reverse-transcribed into cDNA using the QuantiTect Reverse Transcription Kit (Qiagen) in a 20µL reaction, and the cDNA was stored at -20°C until use. Quantitative real-time PCR (qRT-PCR) was performed using an ABI 7500 Real-Time PCR System (Applied Biosystems, Foster City, CA, USA) to assess mRNA expression levels of Star, Cyp11a1, and Hsd17b3. The forward and reverse primer sequences for each gene are provided in Table 1.

**Table 1:** Forward and reverse primer sequences for the evaluated genes

Gene (Accession No.)	Primer Direction	Sequence (5′→3′)	Product Length (bp)
Star (NM_031558.3)	Forward	AGATGAAGTGCTAAGTAAG	150
	Reverse	TTGATTTCCTTGACATTTG	
Cyp11a1 (NM_017286.3)	Forward	AAAGTATCCGTGATGTGGG	112
	Reverse	TTTCTGGGCATAGTTGAGC	
Hsd17b3 (NM_054007.1)	Forward	ATTACCTCCGTAGTCAAGA	176
	Reverse	TATTCCACATTCAAAGCCT	
GAPDH (NM_017008.4)	Forward	AAGAAGGTGGTGAAGCAGGCATC	112
	Reverse	CGAAGGTGGAAGAGTGGGAGTTG	

Each 20 µL reaction contained 10 µL SYBR Green Master Mix (Qiagen), 0.5 µM of each primer, and 2 µL cDNA. Cycling conditions were: 95°C for 10 min, followed by 40 cycles of 95°C for 15 s and 60°C for 1 min. Amplification specificity was confirmed by melting curve analysis, and primer efficiency was validated using standard curve slopes. Gene expression was normalized to GAPDH and calculated using the 2-∆∆Ct method (Koohpeyma *et al*., 2022).

### Stereological Analysis

#### Testicular Preparation and Sectioning

The testes were excised, immersed in isotonic saline to measure initial weight and volume, and fixed in 10% neutral-buffered formalin. Using the orientator method, each testis was cut into 8--12 slabs to generate isotropic uniform random (IUR) sections. Circular tissue samples were punched out with a trocar and embedded in paraffin blocks together with the remaining slabs. Paraffin-embedded tissues were sectioned at 5µm (for volume estimation) and 20 um (for cell counting). Sections were stained with hematoxylin-eosin according to established protocols. Post-embedding volume and the tissue-shrinkage coefficient (d_shr) were calculated as: follows, where AA is the area after processing, and AB is the area before processing (Koohpeyma *et al*., 2024).


$$\text{ Volume Shrinkage }=1-(\mathrm{AA}/\mathrm{AB}{)}^{1.5}$$

### Volume Estimation of Interstitial Tissue and Seminiferous Tubules

In 5µm sections, the volume density (Vv) of the seminiferous tubules and interstitial tissue was determined using the point-counting method and Delesse’’s principle:


$$\text{ Vv structure }=\frac{\sum_{i=1}^{n}p\text{ structure }}{\sum_{i=1}^{n}p\text{ reference }}$$

The absolute volume of each structure was calculated as follows:


$$V_{\text{(testis) }}\times Vv_{\text{(structure) }}=V_{\text{(structure) }}$$

where ∑p(structure) is the number of points over the structure, and ∑p(reference) is the total number of test points (Koohpeyma *et al*., 2024).

### Length, Diameter, and Height of the Germinal Epithelium

The length density (Lv) of the seminiferous tubules was estimated using a test frame at 180x magnification.


$$Nv=\frac{\sum_{i=1}^{n}Q}{\sum_{i=1}^{n}p\times h\times \left(\frac{a}{f}\right)}\times \frac{t}{BA}$$

where ∑Q is the number of tubule profiles counted, a/fa is the frame area, and ∑F is the number of frames analyzed. Total length (L) was calculated as Lv x total tubule volume. Tubule diameter was measured in 110--130 tubules per sample, by recording the maximum perpendicular diameter across the long axis. Germinal epithelium height was determined in 5 µm sections from 8-10 fields using systematic random sampling:


$$H=\frac{Vv}{Sv}$$

where Vv is the volume density, and Sv is the surface density of the germinal epithelium (Koohpeyma *et al*., 2024).

### Quantification of Testicular Cell Populations

The optical dissector method was used to estimate the number of testicular cells (spermatogonia, spermatocytes, round and elongated spermatids, Sertoli cells, and Leydig cells) in 20µmsections. Numerical density (Nv) was calculated as follows (Koohpeyma *et al*., 2024):


$$Nv=\frac{2\times \sum_{i=1}^{n}Q}{\sum^{F}p\times \left(\frac{BA}{af}\right)t}$$

where ΣQ is the number of nuclei counted, h is the dissector height, a/f is the counting -frame area, ΣP is the number of fields, t is the mean section thickness, and BA is the microtome block advance. Total cell numbers were calculated as Nv ×x reference volume. For sexual -lineage cells (Koohpeyma *et al*., 2024):


$$V_{\text{(seminiferous tubule/Interstitial tissue) }}\times Nv_{\text{(sexual lineage cells) }}=N_{\text{(sexual lineage cells) }}$$

### Sperm Analysis

#### Epididymal Sperm Collection

Spermatozoa were collected from the caudal epididymis through a 2 mm incision, suspended in 5 mL of normal saline at 37°C, and gently agitated for 5 min to ensure uniform distribution (Oladele *et al*., 2022).

### Sperm Motility

A 10 µL sperm suspension was placed on a pre-warmed slide (37°C), covered with a coverslip, and examined at 400x magnification using a light microscope (Nikon E-200, Japan). Motility was assessed in 10 random fields per animal and categorized according to WHO (2010) guidelines as rapid progressive, slow progressive, non-progressive, or immotile.

### Sperm Count

Sperm concentration was measured using a Neubauer hemocytometer (LABART, Germany; depth, 0.1 mm) at 40x magnification. Sperm were counted in four large squares, and concentration was expressed as x10^6^ sperm/mL after adjustment for dilution (Oladele *et al*., 2022).

### Sperm Morphology

Sperm smears were stained with 1% eosin (Merck, Darmstadt, Germany) for 5-10 min and air-dried. Morphological abnormalities (e.g., amorphous heads, double tails, bent tails) were evaluated in 200-300 spermatozoa per sample at 400× magnification (Oladele *et al*., 2022).

### Sperm Viability

Viability was assessed using eosin-nigrosine staining (Shati, 2019). Sperm suspension was mixed with 1% eosin Y (1:2 v/v) for 30 s, followed by an equal volume of nigrosine (Merck). Smears were examined at 400× magnification. Live spermatozoa remained unstained, while non-viable spermatozoa appeared red.

### Statistical Analysis

All statistical analyses were performed using SPSS software (version 23; SPSS Inc., Chicago, IL, USA). The Kolmogorov-Smirnov test was first used to assess data normality. As the data were normally distributed and variances were homogeneous, parametric tests were employed. Differences among the means of the four study groups (control, CGA, BUS, and BUS+CGA) were evaluated using one-way analysis of variance (ANOVA), followed by Tukey’’s posthoc test. Data are expressed as mean±standard deviation (SD), and *p*<0.05 was considered statistically significant.

## RESULTS

### Hormonal Levels and Oxidative Stress

**Figure 1:**
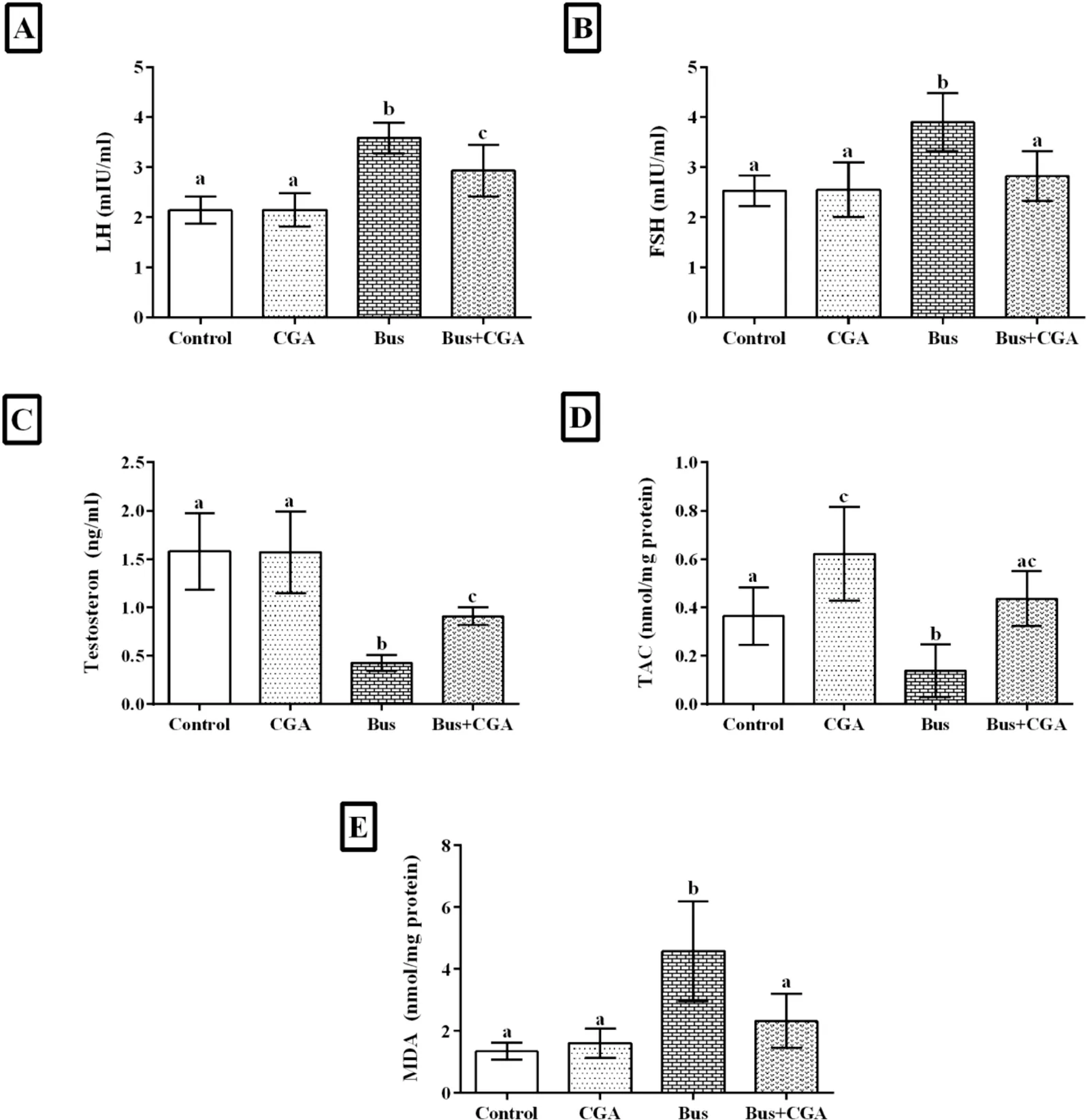
Evaluation of serum concentrations of luteinizing hormone (LH), follicle-stimulating hormone (FSH), and testosterone, as well as total antioxidant capacity (TAC) and malondialdehyde (MDA) levels in the experimental groups. Data are expressed as mean±SD. Statistical analysis was performed using Tukey’s post-hoc test. Columns marked with identical superscript letters (a, b, or ab) indicate no significant differences between those groups (*p*≥0.05), whereas different superscript letters indicate statistically significant differences (*p*<0.05).

BUS treatment significantly increased serum LH and FSH levels and reduced serum testosterone levels compared with the control group (*p*<0.05; Figure 1A-C). In contrast, administration of CGA alone showed no significant differences in these hormone levels compared with the control group (*p*>0.05). Co-administration of BUS+CGA significantly increased testosterone levels compared with the BUS group (*p*<0.05), although they remained significantly lower than those in the controls (*p*<0.05). Regarding oxidative stress, BUS significantly decreased TAC and increased MDA levels in testicular tissue, indicating enhanced lipid peroxidation and oxidative damage (*p*<0.05; Figure 1D-E). In the BUS+CGA group, CGA treatment significantly increased TAC and reduced MDA levels compared with the BUS group (*p*<0.05), approaching control values (*p*>0.05 *vs*. control). The CGA group showed no significant changes in MDA levels compared with controls (*p*>0.05). These findings suggest that CGA mitigates busulfan-induced hormonal disruption and oxidative stress through its antioxidant properties.

### Expression of Steroidogenic Genes (*STAR, CYP11A1, HSD17B3*)

**Figure 2:**
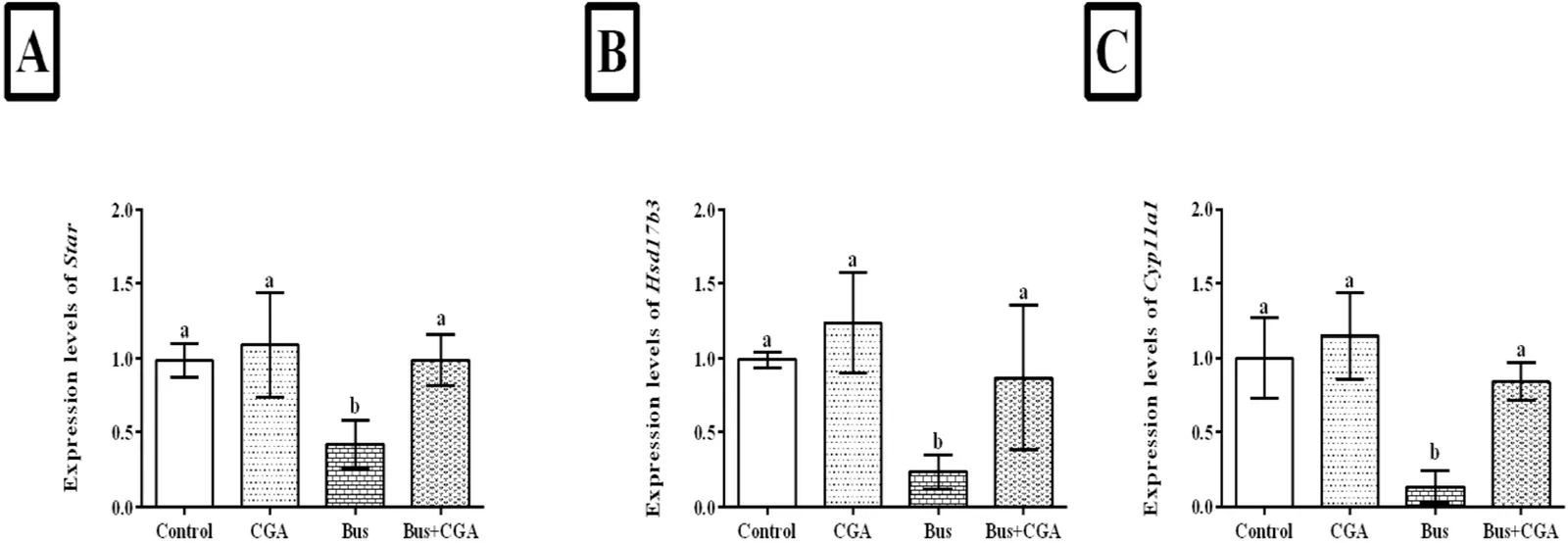
mRNA expression levels of (A) STAR, (B) HSD17B3, and (C) CYP11A1 in rat testicular tissue across experimental groups after 60 days of treatment. Data are presented as mean±SEM. Based on the Tukey post-hoc test, groups with the same superscripts (a, b) show no significant difference (*p*≥0.05), while different superscripts indicate significant differences (*p*<0.05).

As shown in Figure 2A-C, BUS treatment significantly reduced the mRNA expression of STAR, CYP11A1, and HSD17B3 in testicular tissue compared with the control group (*p*<0.05). Relative controls (normalized to 1.0±0.1 fold-change using the 2-∆∆Ct method), the BUS group showed reduced expression levels of STAR (0.5±0.1), CYP11A1 (0.5±0.1), and HSD17B3 (0.5±0.1). The CGA group exhibited no significant differences in gene expression compared with controls (*p*>0.05). In the BUS+CGA group, CGA treatment significantly increased the expression of STAR (1.0±0.1), CYP11A1 (1.0±0.1), and HSD17B3 (1.0±0.1) compared with the BUS group (*p*<0.05), with no significant difference from the controls (*p*>0.05). These results indicate that CGA ameliorates busulfan-induced downregulation of steroidogenic genes, thereby supporting testosterone biosynthesis and spermatogenesis.

### Stereological Parameters of the Testis

#### Testis Weight and Volume

**Figure 3:**
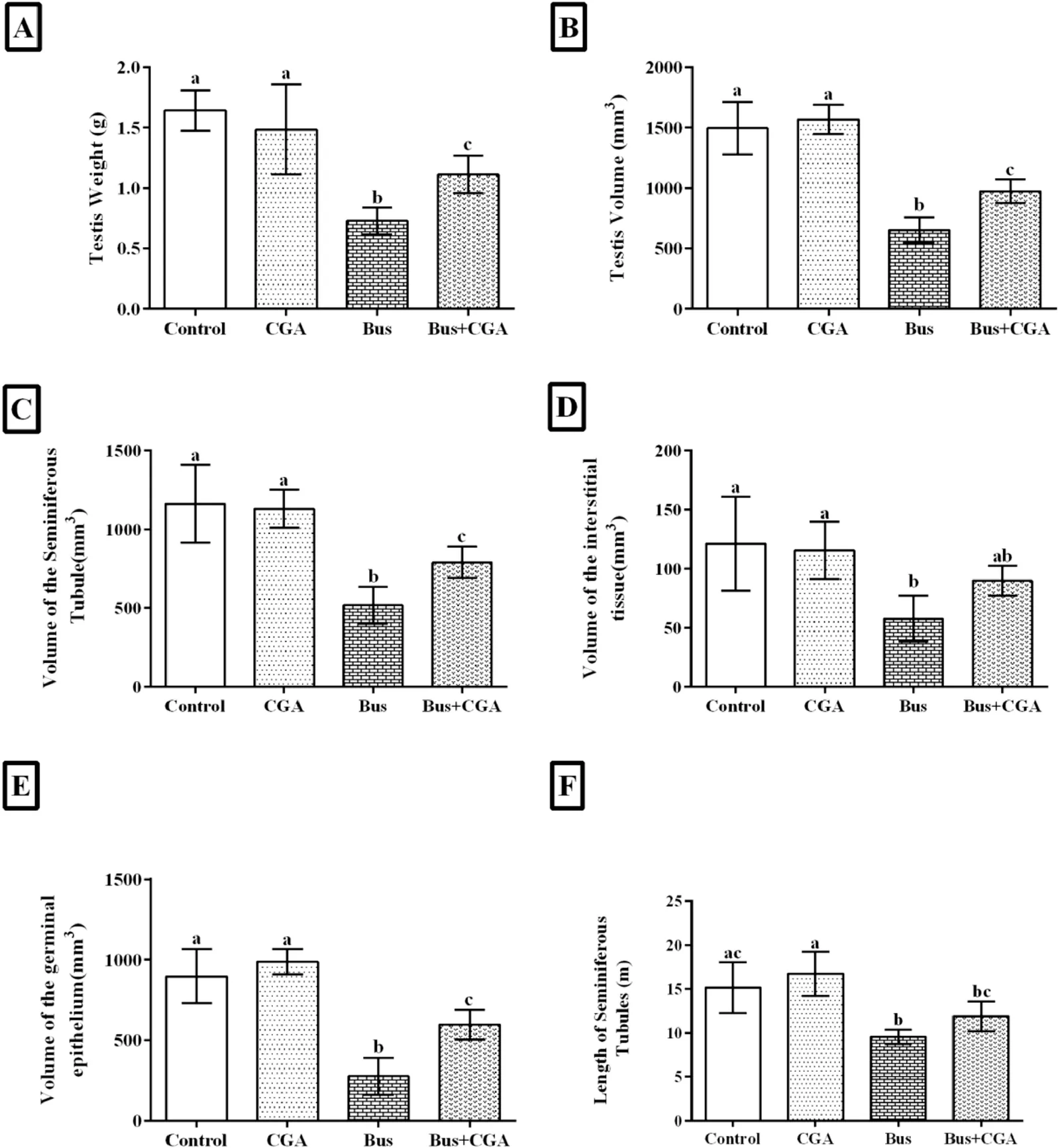
Evaluation of stereological parameters of the testis after 60 days of treatment. (A) Testis weight, (B) Testis volume, (C) Volume of the seminiferous tubules, (D) Volume of the interstitial tissue, (E) Volume of the germinal epithelium, and (F) Length of the seminiferous tubules in the experimental groups (Control, CGA, BUS, and BUS+CGA). Data are presented as mean ± SD. According to the post-hoc Tukey test, groups with the same superscripts (a, b, c, ab, ac, bc) are not significantly different (*p*≥0.05), while dissimilar letters indicate a statistically significant difference (*p*<0.05).

Stereological analysis revealed significant busulfan-induced alterations in testicular structure (Figure 3A-F). The BUS group exhibited significant reductions in testis weight, testis volume, seminiferous tubule volume, germinal epithelium volume, interstitial tissue volume, and seminiferous tubule length compared with the control group (*p*<0.05). These changes reflect severe testicular atrophy and disrupted spermatogenic architecture. The CGA group showed no significant differences in these parameters compared with controls (*p*>0.05), indicating that CGA had no adverse effects on testicular structure. In the BUS+CGA group, co-treatment with CGA significantly improved all stereological parameters compared with the BUS group (*p*<0.05), although the values remained significantly lower than those in the controls (*p*<0.05). These findings suggest that CGA partially restores testicular morphology after busulfan-induced damage.

### Germ and Somatic Cell Populations

**Figure 4:**
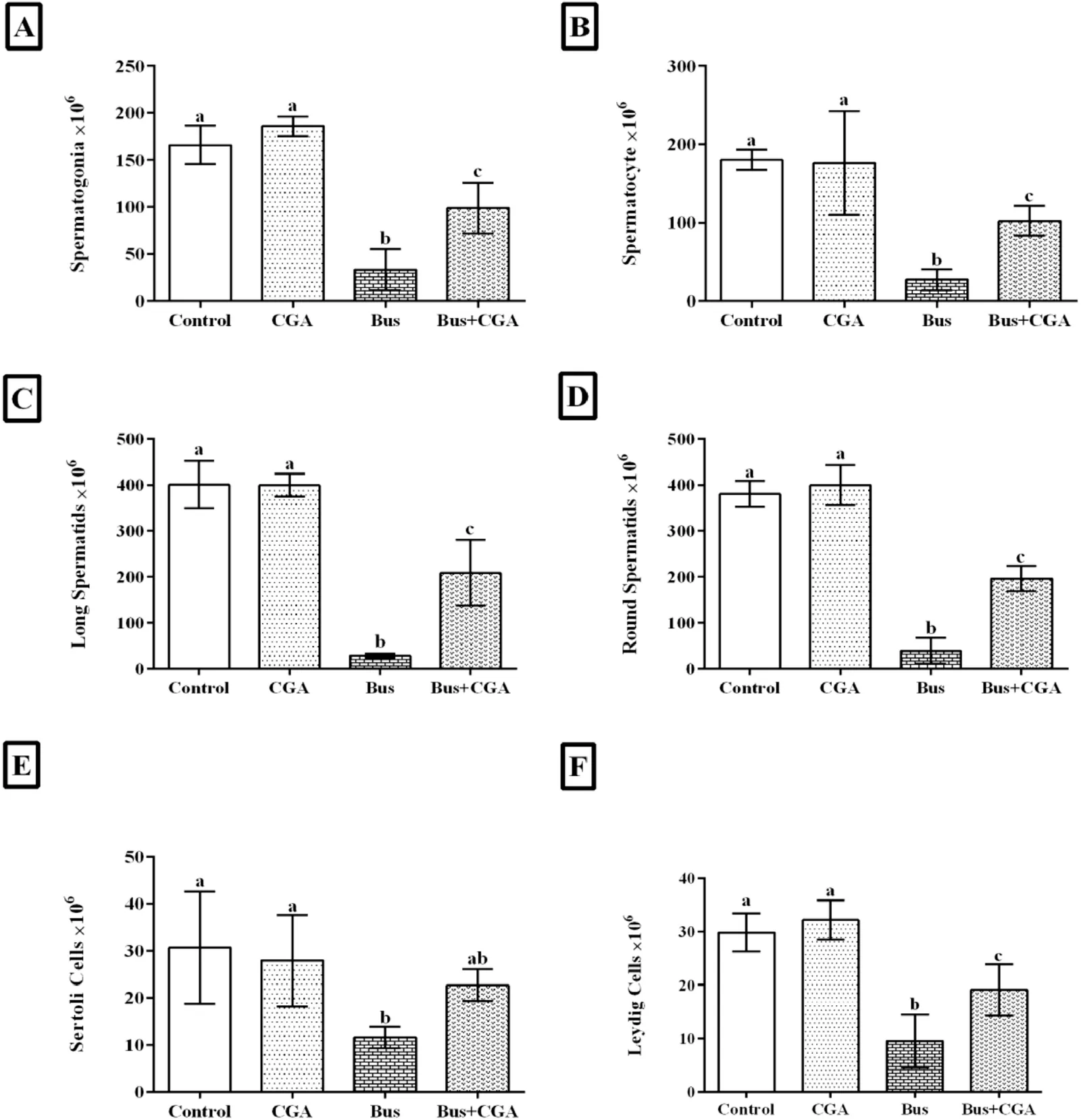
Evaluation of the stereological parameters of the testis after 60 days of treatment. (A) Number of spermatogonia, (B) Spermatocytes, (C) Elongated spermatids, (D) Round spermatids, (E) Sertoli cells, and (F) Leydig cells in the experimental groups. Data are presented as mean±SD. According to the post-hoc Tukey test, groups sharing the same superscripts (a, b, c, d, ab, ac, bc) are not significantly different at α=0.05 (*p*≥0.05). However, dissimilar letters indicate statistically significant differences (*p*<0.05).

BUS treatment significantly reduced the numbers of germ cells (spermatogonia, primary spermatocytes, round spermatids, and elongated spermatids) and somatic cells (Sertoli and Leydig cells) compared with controls (*p*<0.05; Figure 4A-F). The CGA group showed no significant changes in cell populations compared with controls *(p*>0.05). In the BUS+CGA group, CGA treatment significantly increased the numbers of spermatogonia, spermatocytes, round and elongated spermatids, Sertoli cells, and Leydig cells compared with the BUS group (*p*<0.05), although these values were not fully restored to control levels (*p*<0.05). These results demonstrate that CGA mitigates busulfan-induced cellular damage, thereby supporting the preservation of germ and somatic cell populations critical for spermatogenesis.

### Histopathological, Morphometric, and Sperm Parameter Analyses

#### Morphometric Analysis and Sperm Parameters

**Figure 5:**
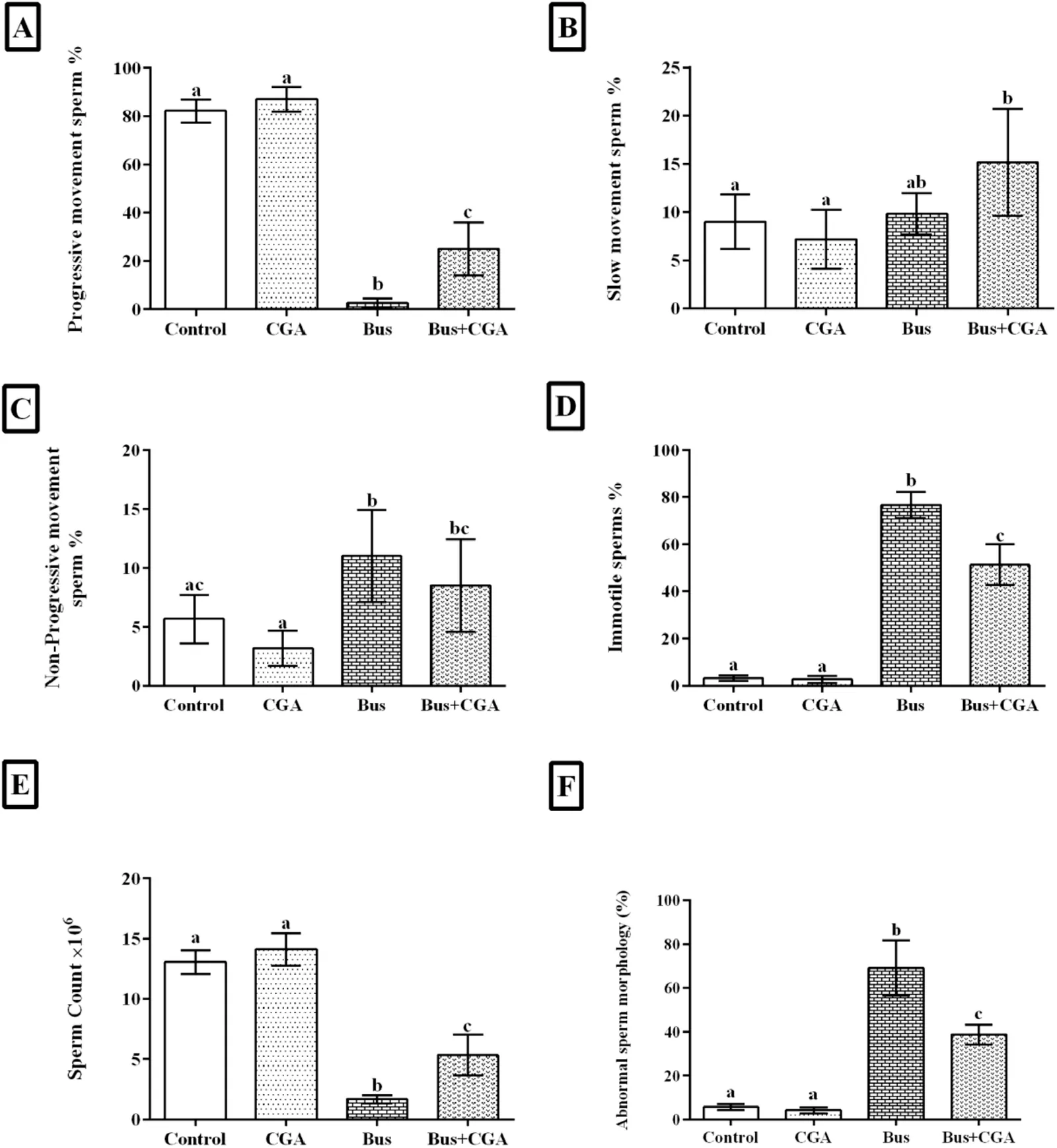
Sperm motility, count, and morphology in the experimental groups. The column graphs represent (A) sperm count, (B) percentage of morphologically normal sperm, and (C) percentage of motile sperm after treatment. Data are expressed as mean±SD. According to the post-hoc Tukey test used for intergroup comparisons, groups with the same superscripts (b, c, d) are not significantly different at α=0.05 (*p*≥0.05). However, dissimilar letters indicate a statistically significant difference (*p*<0.05).

Figure 5A-F presents the morphometric analysis and sperm parameters in the experimental groups. Morphometric analysis revealed that BUS treatment significantly reduced the numbers of round and elongated spermatids, Sertoli cells, and Leydig cells compared with the control group (*p*<0.05), indicating severe testicular damage. In the CGA group, seminiferous tubule diameter and germinal epithelium height were similar to those in the control group, whereas the seminiferous tubule lumen diameter remained unchanged, suggesting no adverse effect of CGA alone. However, in the BUS+CGA group, these morphometric parameters were restored to levels comparable to those of the control group, indicating a protective effect of CGA against busulfan-induced damage (*p*≥0.05 *vs*. control).

Regarding sperm parameters, the control group exhibited baseline motility and sperm count. BUS significantly decreased sperm count (*p*<0.05), consistent with azoospermia or a severe reduction in sperm production. In contrast, the CGA group showed significantly higher motility and sperm production than the control group (*p*<0.05), with a slight reduction in sperm abnormalities. The BUS+CGA group demonstrated a significant increase in sperm count compared with the BUS group (*p*<0.05), although motility remained lower than that of the control group. In the BUS+CGA group, CGA did not further improve motility and may have slightly reduced it compared with the control group (*p*<0.05). Sperm abnormalities were markedly higher in the BUS group, but pretreatment with CGA significantly reduced abnormalities and improved motility compared with BUS-treated animals (*p*<0.05).

### Histological evaluation of testicular tissue

**Figure 6:**
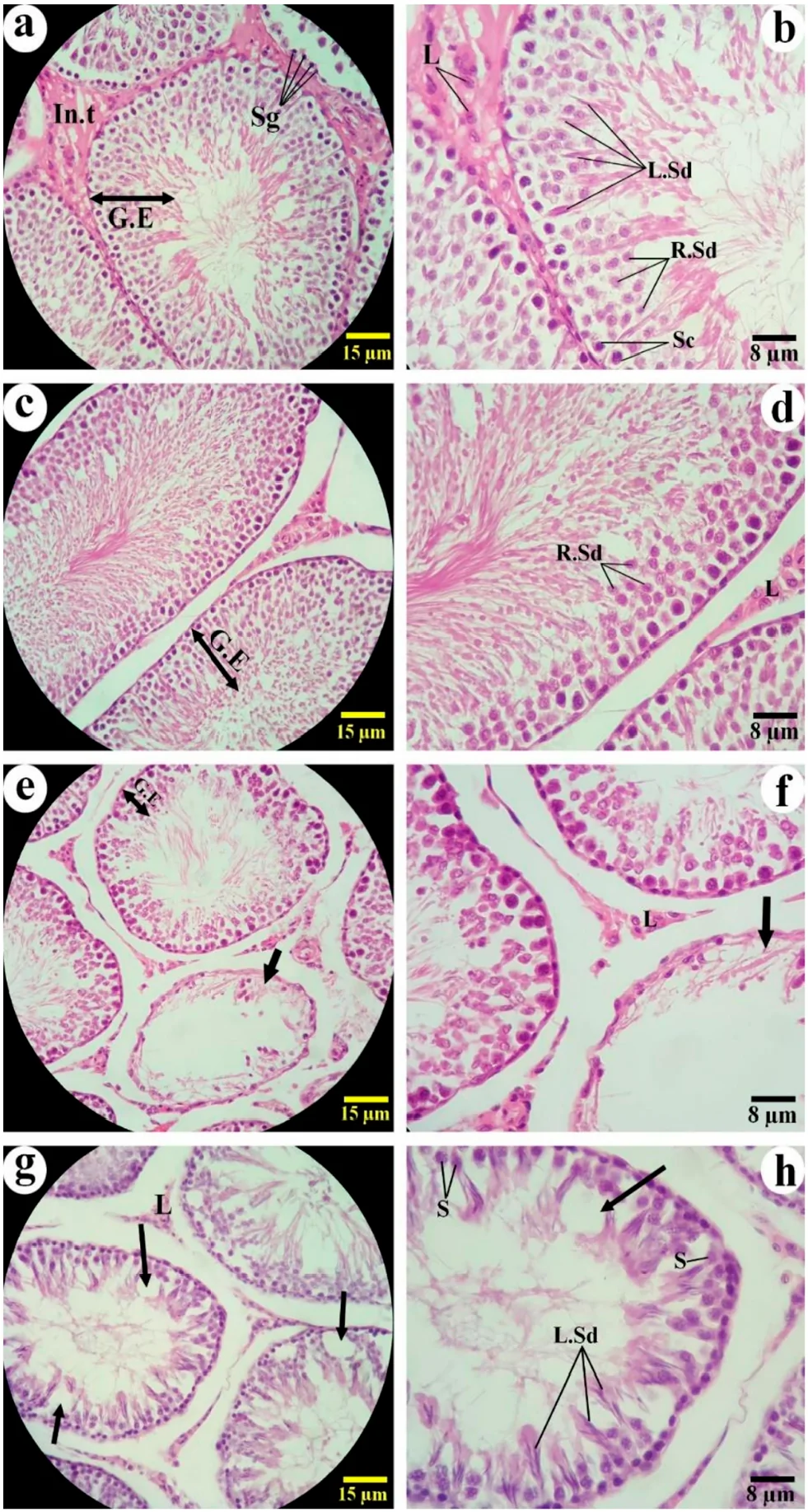
Histological evaluation of testicular tissue using H&E staining in experimental groups (Magnification ×100, ×400, n=6). Panels (a) and (b): Control group; (c) and (d): Chlorogenic acid (CGA)-treated group; (e) and (f): Busulfan-treated group; (g) and (h): Busulfan + CGA co-treated group. Large arrows indicate seminiferous tubules devoid of germ cells, while small arrows show focal areas lacking cellular components within the seminiferous epithelium. Cell types are labeled as follows: Spermatogonia (Sg), Spermatocytes (Sc), Round Spermatids (R.Sd), Elongated Spermatids (L.Sd), Sertoli cells (S), and Leydig cells (L). Hematoxylin and Eosin (H&E) staining was used to visualize histological structure.

Hematoxylin and eosin (H&E) staining revealed distinct histological differences among the experimental groups (Figure 6A-H). In the control group (a, b), the seminiferous tubules displayed normal architecture with intact germinal epithelium (GE), numerous spermatogonia (Sg), spermatocytes (Sc), round spermatids (R.Sd), and elongated spermatids (L.Sd). Sertoli (S) and Leydig (L) cells appeared normal, and the lumen was densely populated with mature germ cells.

In the CGA-treated group (c, d), the seminiferous tubules maintained structural integrity similar to that of the control group, with no evident signs of degeneration or cellular loss.

In contrast, the BUS-treated group (e, f) showed marked histopathological damage, including disorganized germinal epithelium, reduced cellular density, large vacuolization (V), and detachment of spermatogenic cells. Seminiferous tubules appeared atrophic, with focal areas devoid of germ cells (arrows).

In the BUS+CGA co-treated group (g, h), partial restoration of testicular histology was observed. The seminiferous tubules exhibited improved architecture compared with the busulfan-treated group, with increased numbers of germ cells and reduced vacuolization, although they were not fully comparable to those of the control group.

## DISCUSSION

This study demonstrates that chlorogenic acid (CGA) effectively ameliorates busulfan-induced azoospermia in a rat model by enhancing spermatogenesis, steroidogenesis, and testicular architecture through its antioxidant, anti-inflammatory, and anti-apoptotic properties. The gonadotoxic effects of busulfan, characterized by oxidative stress, inflammation, and apoptosis, lead to testicular atrophy, reduced sperm quality, and downregulation of steroidogenic genes (STAR, CYP11A1, and HSD17B3) (Abarikwu *et al*., 2022; Rostami *et al*., 2022; Abd El-Hay *et al*., 2023; Zhao *et al*., 2023; Wu *et al*., 2024). Our findings show that CGA (50 mg/kg for 60 days) significantly counteracted these effects, improving sperm count, motility, hormonal balance, and testicular morphology, thereby positioning CGA as a promising therapeutic agent for fertility preservation in patients receiving chemotherapy.

Busulfan significantly increased oxidative stress, as evidenced by reduced total antioxidant capacity (TAC) and elevated malondialdehyde (MDA) levels in testicular tissue *(p*<0.05), indicating lipid peroxidation and oxidative damage (Eren *et al*., 2020; Abarikwu *et al*., 2022; Wang *et al*., 2025). Co-treatment with CGA restored TAC and reduced MDA levels (*p*<0.05), approaching control values, consistent with its antioxidant properties. These results align with Chen *et al*. (2025), who reported that CGA enhanced superoxide dismutase (SOD), glutathione peroxidase (GPx), and catalase activity while reducing MDA in rats with tripterygium glycoside (TG)-induced asthenozoospermia. Similarly, Demir *et al*. (2025) demonstrated that CGA activated the Nrf2/HO-1 pathway, thereby restoring antioxidant defenses in cisplatin-induced testicular damage (Mentese *et al*., 2023; Demir *et al*., 2025). The polyphenolic structure of CGA facilitates direct ROS scavenging and metal -ion chelation (e.g., iron), thereby inhibiting ROS production through the Fenton reaction (El-Khadragy *et al*., 2021; Kalinowska *et al*., 2022; Mohlala *et al*., 2023; Khan *et al*., 2024). In vitro studies further support the antioxidant effects of CGA by showing reduced ROS levels and enhanced cell viability in testicular cells exposed to oxidative stress (Owumi *et al*., 2021; Abedpour *et al*., 2022). Upregulation of Nrf2 signaling by CGA enhances the expression of antioxidant enzymes such as heme oxygenase-1 (HO-1) and NAD(P)H quinone oxidoreductase 1 (NQO1), thereby protecting testicular tissue from oxidative damage (El-Khadragy *et al*., 2021; Zhang *et al*., 2021; Rotimi *et al*., 2022; Demir *et al*., 2025).

The Nrf2/HO-1 pathway represents a plausible primary mechanism underlying the protective effects of CGA in our model, as it not only neutralizes ROS but also modulates downstream anti-apoptotic and anti-inflammatory genes, directly contributing to the observed restoration of TAC (*p*<0.05) and reduction in MDA levels (*p*<0.05). This pathway likely prevents the oxidative cascade that impairs mitochondrial function in Leydig and germ cells, thereby linking antioxidant defense to the preservation of spermatogenic integrity (Kushwaha *et al*., 2022).

Busulfan-induced hormonal disruptions, including elevated LH and FSH levels and reduced testosterone levels (*p*<0.05), reflect impaired Leydig -cell function and dysregulation of the hypothalamic-pituitary-gonadal axis (Oyovwi, 2023; Zhao *et al*., 2023; Maluin *et al*., 2024; Wu *et al*., 2024). Co-treatment with CGA significantly restored testosterone levels and normalized gonadotropins (*p*<0.05), although not fully to control values. This effect was paralleled by upregulated mRNA expression of STAR, CYP11A1, and HSD17B3 (from 0.5±0.1 to 1.0±0.1 fold-change, *p*<0.05), which facilitate cholesterol transport, pregnenolone synthesis, and testosterone production, respectively (Bamodu *et al*., 2021; Chen *et al*., 2025). These findings are consistent with Chen *et al*. (2025), who reported increased intratesticular testosterone in TG-treated rats through upregulation of steroidogenic genes. Mu *et al*. (2022) further showed that CGA enhances STAR expression through SHP2-mediated signaling, thereby supporting Leydig-cell function. Comparable effects were observed in models of arsenicand tamoxifen-induced reproductive toxicity, in which CGA restored testosterone and gonadotropin levels (El-Khadragy *et al*., 2021; Abedpour *et al*., 2022; Shah *et al*., 2023). Modulation of pathways, such as PI3K/AKT, MAPK, and ERK1/2, by CGA may further enhance steroidogenic gene expression and cell survival, as reported in testicular and diabetic models (Zhang *et al*., 2021; Koohpeyma *et al*., 2022; Komeili-Movahhed *et al*., 2023; Khan *et al*., 2024).

The PI3K/AKT and MAPK/ERK1/2 pathways likely mediate the effects of CGA by inhibiting apoptosis and promoting cholesterol uptake in Leydig cells, thereby explaining the observed upregulation of STAR, CYP11A1, and HSD17B3 (from 0.5±0.1 to 1.0±0.1- fold-change, *p*<0.05). These pathways link antioxidant effects to steroidogenic recovery and provide a mechanistic basis for the role of CGA in ameliorating busulfan-induced hormonal imbalances (Deng *et al*., 2021).

Stereological analyses revealed busulfan-induced testicular atrophy, with significant reductions in testis weight, seminiferous tubule volume, germinal epithelium volume, interstitial tissue volume, tubule length, and the counts of germ cells (spermatogonia, spermatocytes, and spermatids) and somatic cells (Sertoli and Leydigcells) (*p*<0.05) (Hafezi *et al*., 2022; Rostami *et al*., 2022). Co-treatment with CGA significantly improved these parameters (*p*<0.05), although they were not fully restored to control levels, as confirmed by histopathological evaluation showing reduced vacuolization and improved tubular architecture. Morphometric analysis further showed that CGA restored seminiferous tubule diameter and germinal epithelium height to levels comparable to those of the controls (*p*≥0.05). These findings corroborate those of Chen *et al*. (2025), who reported restored seminiferous tubule organization in TG-treated rats, and Demir *et al*. (2025), who observed improved Johnsen scores and spermatogenesis in cisplatin-treated rats. CGA also enhanced sperm count and reduced abnormalities (*p*<0.05), although motility remained lower than in controls, consistent with studies of polyphenol-rich compounds that improved sperm parameters in infertile models (Khalaf *et al*., 2024; Signorini *et al*., 2025). The integration of stereological, histopathological, and molecular data underscores the multifaceted role of CG in repairing testicular microarchitecture and supporting spermatogenesis (Koohpeyma *et al*., 2022).

Although inflammatory markers (e.g., TNF-α, and IL-6) were not measured, the anti-inflammatory properties of CGA likely contributed to its protective effects. Busulfan-induced oxidative stress triggers inflammation, elevating pro-inflammatory (Eren *et al*., 2020; Zhang *et al*., 2021; Komeili-Movahhed *et al*., 2023). Demir *et al*. (2025) reported that CGA reduced NF-κB p65, IL-6, and myeloperoxidase in cisplatin-treated rats, whereas El-Khadragy *et al*. (2021) showed decreased inflammation in arsenic-induced testicular toxicity. CGA also mitigated apoptosis, as evidenced by reduced caspase-3 activity in cisplatin and tamoxifen models (Abedpour *et al*., 2022; Komeili-Movahhed *et al*., 2023; Cortez *et al*., 2024; Demir *et al*., 2025). These anti-inflammatory and anti-apoptotic effects, potentially mediated by SHP2/ERK1/2, PI3K/AKT, or NF-κB pathways, create a favorable microenvironment for spermatogenesis and steroidogenesis (Zhang *et al*., 2021; Mu *et al*., 2022; Shah *et al*., 2023; Khan *et al*., 2024).

The clinical significance of CGA lies in its potential as an adjuvant therapy for fertility preservation in cancer patients undergoing busulfan-based chemotherapy, in whom azoospermia affects up to 90% of survivors and leads to long-term infertility (Atwa *et al*., 2023). Unlike other antioxidants (e.g., vitamin E and N-acetylcysteine), the ability of CGA to improve sperm DNA integrity and hormonal balance suggests that it could enhance outcomes when integrated with sperm banking or cryopreservation protocols, offering a cost-effective strategy for fertility preservation (Signorini *et al*., 2025).

Future directions include evaluating the long-term effects of CGA on fertility outcomes in larger animal models (e.g., non-human primates) to better mimic human physiology and exploring nanotechnology-based delivery systems to enhance its bioavailability and targeted action in testicular tissue (Mohlala *et al*., 2023). Additionally, integrating single-cell RNA sequencing could elucidate cell-specific responses to CGA, complementing protein-level validation by Western blotting or immunohistochemistry for STAR, CYP11A1, and HSD17B3 (Livak & Schmittgen, 2001).

Limitations include the lack of direct measurements of antioxidant enzymes (e.g., SOD and catalase) and inflammatory markers (e.g., TNF-α and IL-6). The 2-∆∆Ct method for gene-expression analysis may be sensitive to reference-gene variability, necessitating validation by Western blotting or immunohistochemistry to confirm protein-level changes in STAR, CYP11A1, and HSD17B3 (Cincotta, 2022).

Future studies should quantify specific antioxidant enzymes, inflammatory cytokines, and signaling pathways (e.g., Nrf2, PI3K/AKT, and NF-κB), as demonstrated in cisplatin and diabetic models (Komeili-Movahhed *et al*., 2023; Demir *et al*., 2025).

## CONCLUSION

CGA significantly ameliorates busulfan-induced azoospermia in rats by enhancing antioxidant defenses, restoring steroidogenesis, and repairing testicular architecture. The upregulation of STAR, CYP11A1, and HSD17B3 (from 0.5±0.1 to 1.0±0.1- fold-change), coupled with improved stereological, histopathological, and sperm parameters, highlights the multifaceted protective effects of CGA. Supported by evidence from TG, cisplatin, and arsenic models, CGA emerges as a promising complementary therapy for fertility preservation in patients receiving chemotherapy, warranting further mechanistic and clinical investigation.

## Abbreviations

(CGA): chlorogenic acid;

BUS): busulfan;

(TGs): tripterygium glycosides;

(Cyp11a1): cytochrome

P450 family 11 subfamily A polypeptide 1;

(ELISA): enzyme-linked immunosorbent assay;

(FSH): follicle-stimulating hormone;

(GnRH): gonadotropin-releasing hormone;

(LH): luteinizing hormone;

(MDA): malondialdehyde;

(ROS): reactive oxygen species;

(Star): steroidogenic acute regulatory protein;

(TAC): total antioxidant capacity;

(Hsd17b3): 17β-hydroxysteroid dehydrogenase-3;

(H&E): hematoxylin and eosin.
